# [Retracted] Molecular hydrogen improves type 2 diabetes through inhibiting oxidative stress

**DOI:** 10.3892/etm.2024.12591

**Published:** 2024-05-29

**Authors:** Yi Ming, Qi-Hang Ma, Xin-Li Han, Hong-Yan Li

Exp Ther Med 20:359–366, 2020; DOI: 10.3892/etm.2020.8708

Following the publication of this paper, it was drawn to the Editor’s attention by a concerned reader that the H&E-stained images shown in Fig. 3D on p. 363 were strikingly similar to data that had already been published in different form in another article written by different authors at a different research institute [Zhang J, Wong MG, Wong M, Gross S, Chen J, Pollock, C and Saad S: A cationic-independent mannose 6-phosphate receptor inhibitor (PXS64) ameliorates kidney fibrosis by inhibiting activation of transforming growth factor-β1. PLoS One 10: e0116888, 2015].

Owing to the fact that the contentious data in the above article had already been published prior to its submission to *Experimental and Therapeutic Medicine,* the Editor has decided that this paper should be retracted from the Journal. The authors were asked for an explanation to account for these concerns, but the Editorial Office did not receive a reply. The Editor apologizes to the readership for any inconvenience caused.

